# Formaldehyde quantification using gas chromatography–mass spectrometry reveals high background environmental formaldehyde levels

**DOI:** 10.1038/s41598-024-71271-z

**Published:** 2024-09-04

**Authors:** Sara Y. Chothia, Vicki L. Emms, Liam A. Thomas, Natasha F. A. Bulman, Paul S. Monks, Rebecca L. Cordell, Richard J. Hopkinson

**Affiliations:** 1 Leicester Institute for Structural and Chemical Biology and School of Chemistry, University of Leicester, Henry Wellcome Building, Lancaster Road, Leicester, LE1 7RH UK; 2 Space Park Leicester, University of Leicester, 92 Corporation Road, Leicester, LE4 5SP UK

**Keywords:** Biological techniques, Chemical biology

## Abstract

Formaldehyde (HCHO) is a human toxin that is both a pollutant and endogenous metabolite. HCHO concentrations in human biological samples are reported in the micromolar range; however, accurate quantification is compromised by a paucity of sensitive analysis methods. To address this issue, we previously reported a novel SPME–GC–MS-based HCHO detection method using cysteamine as an HCHO scavenger. This method showed cysteamine to be a more efficient scavenger than the widely used *O*-(2,3,4,5,6-pentafluorobenzyl)hydroxylamine, and enabled detection of aqueous HCHO in the nanomolar range and quantification in the micromolar range. However, quantification in this range required immersive extraction of the HCHO-derived thiazolidine, while a high background signal was also observed. Following on from these studies, we now report an optimised head-space extraction SPME–GC–MS method using cysteamine, which provides similarly sensitive HCHO quantification to the immersive method but avoids extensive wash steps and is therefore more amenable to screening applications. However, high background HCHO levels were still observed A Complementary GC–MS analyses using a 2-aza-Cope-based HCHO scavenger also revealed high background HCHO levels; therefore, the combined results suggest that HCHO exists in high (i.e. micromolar) concentration in aqueous samples that precludes accurate quantification below the micromolar range. This observation has important implications for ongoing HCHO quantification studies in water, including in biological samples.

## Introduction

Quantifying reactive small molecules in biological samples is often challenging due to their small size and promiscuous reactivity. This is particularly the case for formaldehyde (HCHO), which is an environmental pollutant and human metabolite^1–11^. At high concentrations, HCHO is toxic and carcinogenic via mechanisms involving its reactions with biomolecules^1,12–18^. However, the majority of these reactions are unknown, while it is also unclear how reactions directly lead to function- or disease-relevant effects in human cells.

To define HCHO biology, analytical methods are needed that can detect biological (i.e. aqueous) HCHO and can quantify likely small fluctuations that arise in response to external stimuli (e.g. exposure to HCHO or HCHO releasers) or that occur in disease^19–21^. Such methods also have potential diagnostic applications (e.g. biomarker analysis), although the small sample sizes would likely necessitate highly sensitive HCHO quantification. Development of HCHO quantification methods is challenging; however, mass spectrometry-based approaches offer promise in light of their high sensitivity and their ability to discriminate between molecules of different mass. For HCHO, any detection/quantification method relies on the use of scavenging reagents that can sequester HCHO from other reaction partners in complex mixtures (i.e. cells). Commonly used HCHO scavengers include hydroxylamines^22–27^, hydrazines^28–30^, 1-amino-2-thiols (e.g. cysteamine)^27,31–35^ and recently reported 2-aza-Cope probes, which convert HCHO into other carbonyls^36–39^.

In previous work, we reported a novel HCHO quantification method that combines cysteamine scavenging with solid phase microextraction (SPME) and gas chromatography-mass spectrometry (GC–MS) analysis^27^. Our NMR studies with cysteamine revealed it to be a more efficient scavenger than the widely used *O*-(2,3,4,5,6-pentafluorobenzyl)hydroxylamine (PFBHA), while our GC–MS method enabled detection of HCHO at nanomolar concentrations in buffered aqueous samples and in bacterial cell lysate. However, quantification was only possible in the micromolar range. This relatively high quantification limit was, at least in part, a consequence of the high background signal observed for the HCHO-derived thiazolidine. In addition, optimal quantification was only observed when using an immersive extraction method, where the SPME fibre is immersed into the aqueous sample. Immersive extraction requires extensive wash steps between analyses, and is therefore less attractive than non-immersive methods such as head-space extraction.

**Fig. 1:**
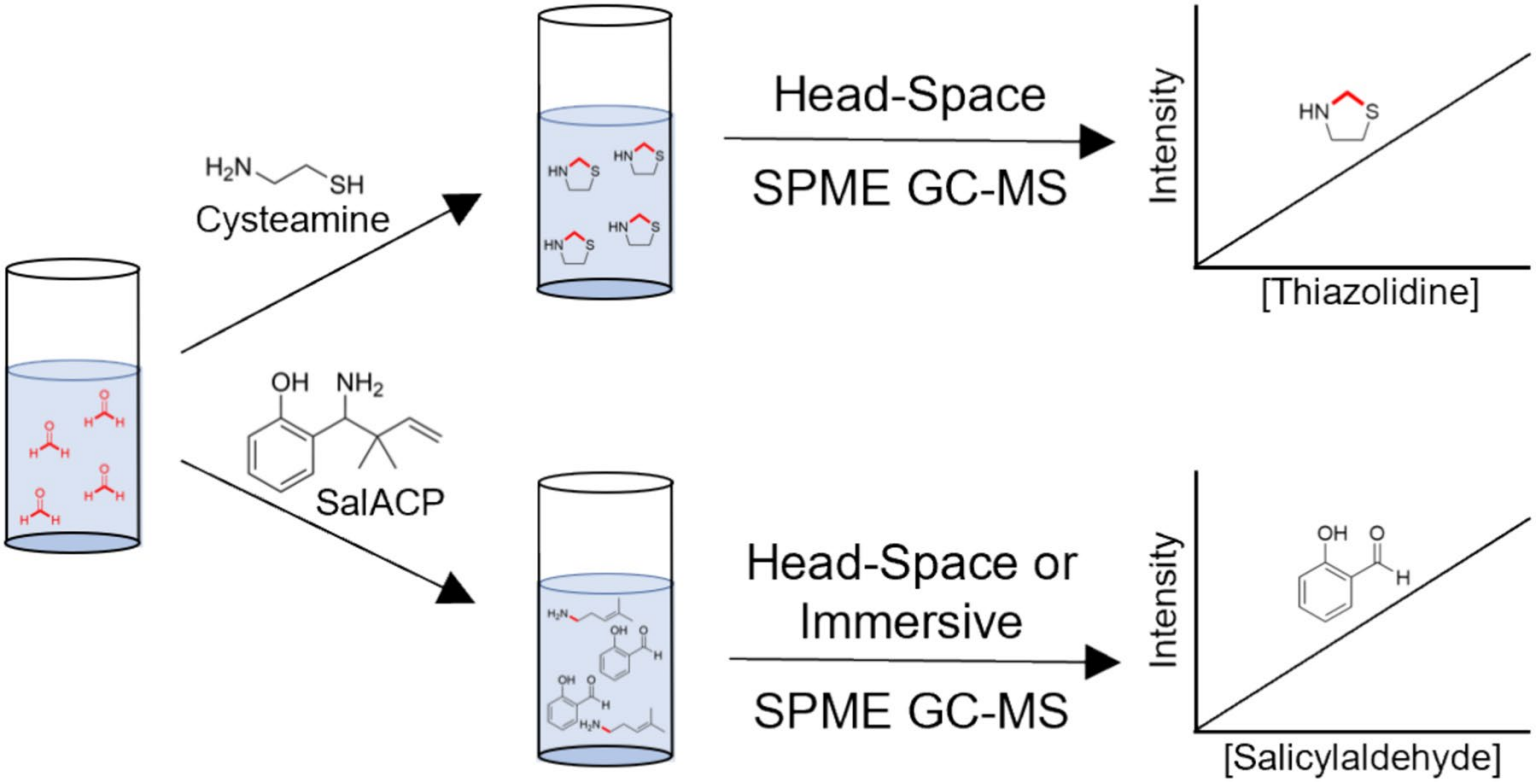
Figure showing HCHO quantification using SPME–GC–MS combined with either cysteamine- or 2-aza-Cope-based HCHO scavenging. After reaction of the scavengers with aqueous HCHO, the resulting products are extracted onto SPME fibres by either head-space or immersive extraction techniques. Desorption from the fibres in the GC mass spectrometer then enables product detection/quantification.

Here, we report development of our SPME–GC–MS methodology with the aim of improving the sensitivity of HCHO quantification (Figs. 1 and S1). These efforts, which used cysteamine or a bespoke 2-aza-Cope HCHO scavenger, resulted in comparably sensitive head-space HCHO quantification to that observed with our previous immersive method. The development of a head-space method should enable faster and more reproducible analyses than immersive methods as it avoids the need for extensive wash steps. However, high background signals were still observed, which precluded HCHO quantification below micromolar levels using either head-space or immersive extraction methods. Therefore, the combined studies suggest that HCHO is a high level (i.e. micromolar) contaminant in our GC–MS quantification studies. Given our use of two different scavengers and multiple sample types and analysis protocols, the results imply that the HCHO derives from the environment rather than the extraction and analysis methods. This result is important because it indicates that environmental HCHO is pervasive and thus prohibits sub-micromolar quantification of HCHO in aqueous samples.

## Results

**Fig. 2:**
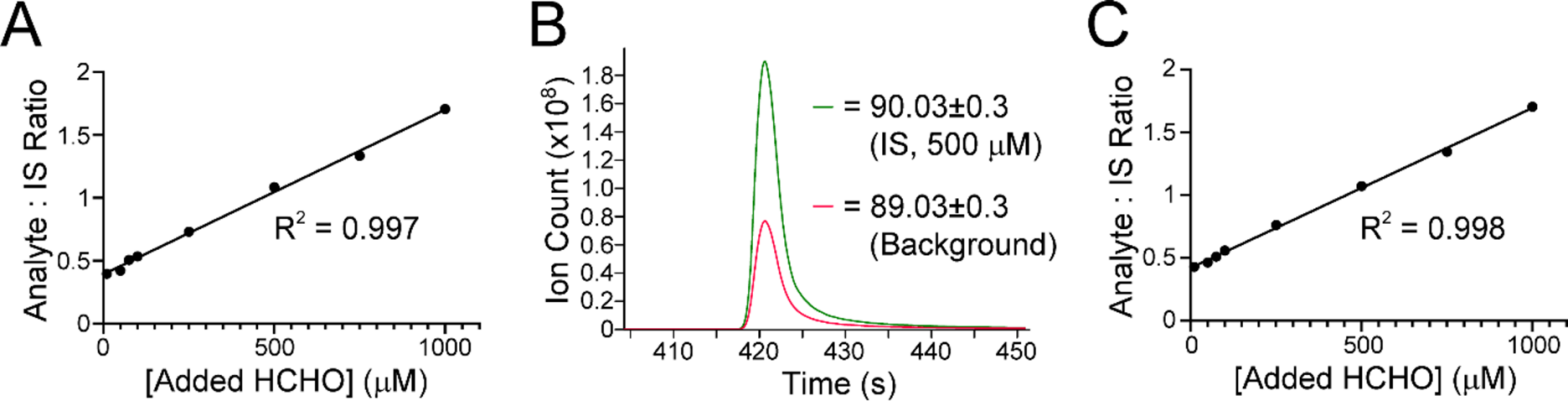
Figure showing cysteamine-based quantification of aqueous HCHO using head-space SPME–GC–MS. (**A**) Graph showing HCHO quantification in 100 mM sodium phosphate buffer pH 7.4. A linear response is observed. (**B**) Image from a GC chromatogram from a buffered sample (100 mM sodium phosphate buffer pH 7.4) without added HCHO after head-space extraction. A peak corresponding to HCHO-derived thiazolidine is observed (red). A peak corresponding to ^13^C-labelled thiazolidine (internal standard, IS) is shown for comparison (green). (**C**) Graph showing HCHO quantification in lysate from HEPG2 cells in 100 mM sodium phosphate buffer pH 7.4. A near-identical response is observed to that in buffer only.

Initial work focused on developing a head-space extraction SPME–GC–MS method using cysteamine as the HCHO scavenger. Our previous head-space method using cysteamine revealed detection of the cysteamine- and HCHO-derived thiazolidine at nanomolar levels^27^. However, titration studies gave a sigmoidal response curve, thus precluding accurate quantification. To improve head-space analysis, we attempted to optimise the procedure by (i) changing to larger sample vials to accommodate a larger sample volume and thus produce higher head-space concentrations, and (ii) transferring to analysis with a newer mass spectrometer (Leco Pegasus BT) with superior sensitivity to our previously used spectrometer (Agilent 5977B). Control studies in buffered water (100 mM sodium phosphate buffer pH 7.4) containing cysteamine (2 mM) and HCHO (10 µM–1 mM) revealed a linear response curve between 50 µM and 1 mM, which is comparable to our previous immersive method (R^2^ = 0.997, Fig. 2A). However, a signal corresponding to the thiazolidine (signal-to-noise ratio > 3) was observed in the samples without added HCHO, while the signal intensity was within 2.5% of that observed with 10 µM HCHO (Fig. 2B). We then attempted to analyse HCHO in buffered lysate from human HEPG2 liver cells. Titration of HCHO into the lysate resulted in a near-identical dose response to that observed in the buffered water samples (R^2^ = 0.998, Fig. 2C), while analysis without added HCHO revealed signals corresponding to the thiazolidine (Fig. S2). However, the intensities of the thiazolidine signals were not significantly different to those observed in buffered water. Overall, the studies with cysteamine suggest that our head-space SPME–GC–MS method is capable of detecting residual HCHO but cannot provide accurate quantification below micromolar concentrations.

**Fig. 3:**
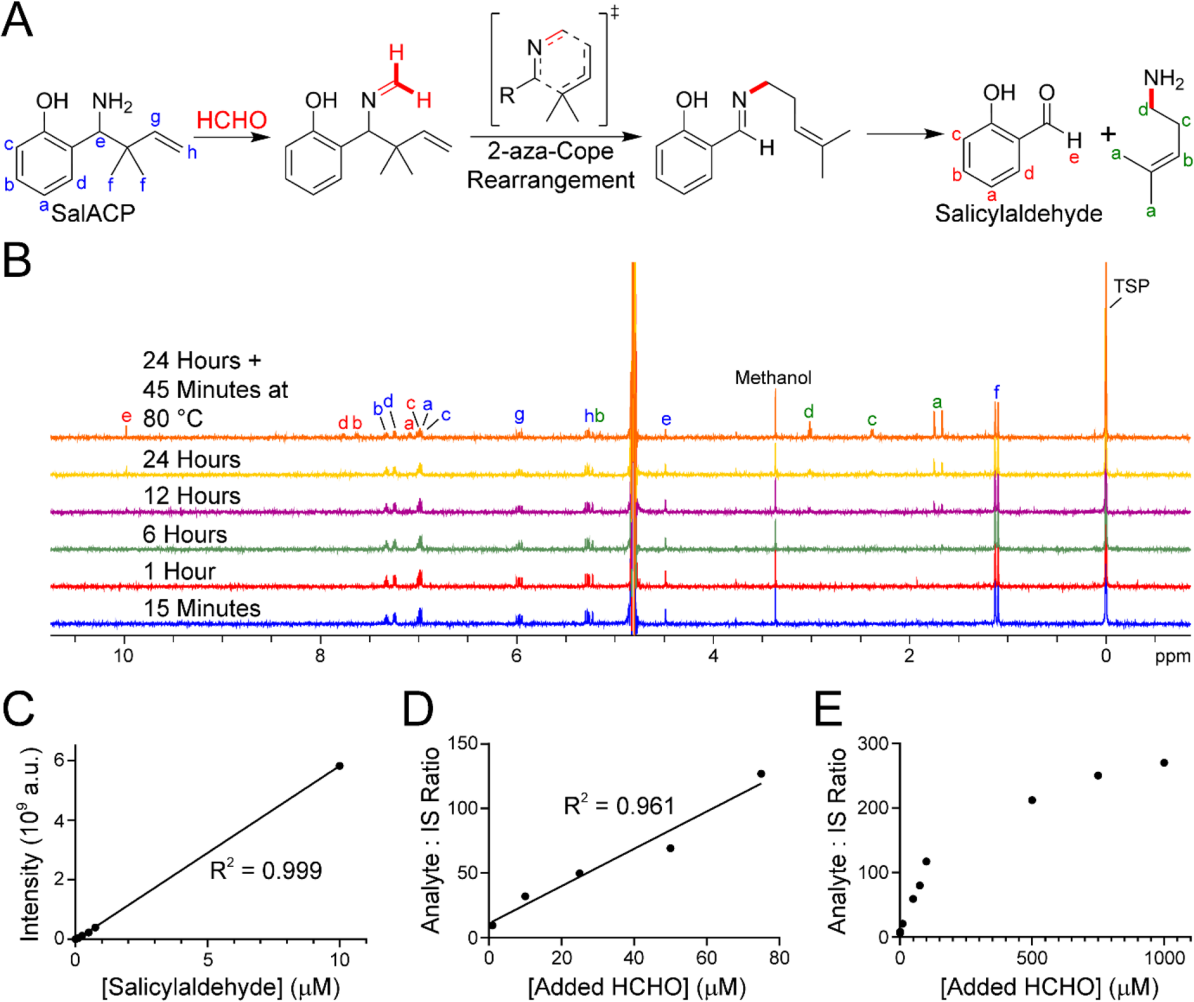
Figure showing SalACP-mediated quantification of aqueous HCHO. (**A**) Scheme showing the reaction of SalACP with HCHO. Following initial imine formation, a 2-aza-Cope rearrangement occurs to form an imine intermediate. Subsequent hydrolysis gives a primary amine and salicylaldehyde, which can be detected using GC–MS. All steps in the reaction are reversible, while it is probable that the rearrangement is accelerated by protonation on the nitrogen. (**B**) ^1^H NMR spectra showing time-dependent formation of salicylaldehyde from the reaction of SalACP (200 µM) and HCHO (100 µM) in buffered water. ^1^H resonances corresponding to the amine product are also apparent. (**C**) Graph showing quantification of salicylaldehyde in 100 mM sodium phosphate buffer pH 7.4 using head-space SPME–GC–MS. A linear response is observed down to 1 nM (R^2^ = 0.999). (**D**) Graph showing quantification of salicylaldehyde derived from the reaction of SalACP and HCHO in 100 mM sodium phosphate buffer pH 7.4 using head-space SPME–GC–MS. A linear response is observed between 1 µM and 75 µM (R^2^ = 0.961). (**E**) Graph showing quantification of salicylaldehyde derived from the reaction of SalACP and HCHO in 100 mM sodium phosphate buffer pH 7.4 using immersive SPME–GC–MS. A linear response is observed between 10 µM and 100 µM (R^2^ = 0.985). Saturation is observed above 100 µM.

Having identified thiazolidine signals in samples untreated with HCHO, we then attempted to detect/quantify HCHO using a different scavenging reagent. For these experiments, we chose to take advantage of 2-aza-Cope-based scavengers, which have been previously used to detect HCHO in cells by fluorescence and chemiluminescence. 2-Aza-Cope probes contain a 1-amino-but-3-ene motif that reacts with HCHO via the amine, forming an imine/iminium. This intermediate then undergoes a [3, 3]-sigmatropic rearrangement, which, after hydrolysis, produces new amino-alkene and carbonyl products that are potentially amenable to quantitative analyses (Fig. 3A). For the SPME–GC–MS studies, we used a previously unreported 1-amino-but-3-ene (SalACP) that we designed to release salicylaldehyde after reaction with HCHO. Initially, the reaction between SalACP and HCHO was monitored by ^1^H NMR, which would provide kinetic parameters and would enable the determination of reaction products. Time-course analyses (over 24 h at 25 °C) of SalACP (200 µM) and HCHO (100 mM) in 100 mM sodium phosphate buffer pH 7.4 (10% v/v D_2_O) revealed a slow reaction but sufficient formation of salicylaldehyde for quantitative analyses (Fig. 3B). Heating the sample to 80 °C for 45 min after 24 h at 25 °C increased salicylaldehyde formation. ^1^H NMR time-course experiments with acetaldehyde in place of HCHO revealed a strong preference for reaction with HCHO, which implies that SalACP is selective for HCHO over longer chain aliphatic aldehydes (Fig. S3).

The head-space SPME–GC–MS method was then used to detect and quantify salicylaldehyde. Initial control samples containing salicylaldehyde (1 pM – 100 µM) in 100 mM sodium phosphate buffer pH 7.4 revealed a linearly quantifiable range down to 1 nM (R^2^ = 0.999, Fig. 3C). Signals corresponding to salicylaldehyde were observed in untreated samples, although their intensities were markedly lower than the intensities observed with 1 nM salicylaldehyde (Fig. S4). Samples of SalACP (200 µM) and HCHO (1 nM – 10 µM) in 100 mM sodium phosphate buffer pH 7.4 were then analysed under the same conditions. However, while salicylaldehyde signals were observed, no dose-dependent response was apparent across the HCHO concentration range (provisional concentrations between 1.9 µM and 2.6 µM based on comparison with the acetophenone internal standard, Fig. S5). Analysing samples containing a fixed concentration of HCHO (500 nM) and varying concentrations of SalACP revealed a linear dose-dependent response between 1 nM (the lowest concentration tested) and 10 µM (R^2^ = 0.999, Fig. S6). ^1^H NMR studies on SalACP pre-heated in buffer revealed no salicylaldehyde formation, which implies that SalACP is stable to the head-space SPME–GC–MS analysis conditions. Therefore, it appears that salicylaldehyde formation is due to reaction with residual HCHO present in the samples (note: residual HCHO-dependent salicylaldehyde formation would not be observable in the ^1^H NMR experiments due to low sensitivity). Repeating the head-space SPME–GC–MS experiment with a fixed SalACP concentration (200 µM) and a higher HCHO concentration range revealed a linear response between 1 µM and 75 µM HCHO (R^2^ = 0.961, Fig. 3D), while a similar linear response (between 10 µM and 100 µM HCHO) was observed using immersive extraction (R^2^ = 0.985, Fig. 3E). Collectively, the SPME–GC–MS analyses with SalACP suggest that residual HCHO in the samples precludes quantification below micromolar concentrations, as observed in our studies with cysteamine.

## Discussion

Quantification of HCHO in aqueous mixtures is essential for ongoing biological studies but is hindered by a lack of sensitive analysis methods. Our studies show that SPME–GC–MS analysis coupled with either cysteamine- and 2-aza-Cope-based HCHO scavenging enables quantification of aqueous HCHO in the micromolar range. Both these methods show comparable sensitivity to most reported HCHO quantification methods^27^ and importantly use scavengers that display a degree of selectivity for HCHO and produce stable and detectable reaction products. These factors are important for HCHO quantification methods in complex mixtures such as biological samples as these samples are small and are likely to contain other reactive carbonyl species. However, despite using two chemically different scavengers and either head-space or immersive extraction methods (which involve different incubation and heating times), we were unable to quantify HCHO at lower concentrations owing to the presence of background signals in the mass spectra. While it is possible that some of this residual HCHO arises from the analysis methods (e.g. leaching from the SPME fibre and/or sample vials and spectrometer components), the fact that HCHO must react with the aqueous scavengers before analysis, in addition to our use of different fibres and different sample preparation and analysis conditions, implies that most/all of the residual HCHO derives from the environment. This observation is important because it suggests that aqueous HCHO quantification is limited by background (even in purified water), thus precluding analysis of biological samples without applying spatiotemporal control (e.g. quantitative live cell imaging) or additional derivatisation that can distinguish between endogenous and environmental HCHO. Optimising such approaches should form the basis of future research aimed at improving biological HCHO quantification methods.

## Methods

### Reagents

All media and buffers were prepared using typical procedures. HCHO stocks were prepared from paraformaldehyde, which was cracked in milliQ water by heating at 100 °C. GC–MS studies with cysteamine used a ^13^C-labelled thiazolidine internal standard, which was prepared by reacting cysteamine (20 mM, Alfa Aesar) with ^13^C-labelled HCHO (10 mM, Santa Cruz Biotechnology) in milliQ water. All other reagents were purchased from the following commercial suppliers: sodium phosphate monobasic monohydrate (Insight Biotechnology), sodium phosphate dibasic anhydrous (MP Biomedicals), D_2_O (Cambridge Isotope Laboratories), DMSO-d_6_ and (Cambridge Isotope Laboratories), chloroform-d_3_ (Cambridge Isotope Laboratories), 3(trimethylsilyl)propionic-2,2,3,3-d_4_ acid sodium salt (TSP, Alfa Aesar), HEPG2 cells (ATCC), HEPG2 cell media (MEM with Earle’s salts, sodium bicarbonate and L-glutamine, Merck), foetal bovine serum (Merck), Pen-Strep (Merck) salicylaldehyde (Merck), acetophenone (Acros), 3-Methyl-2-butenylboronic acid pinacol ester (Merck), caesium carbonate (Acros), methanol (Fisher), tetrahydrofuran (Fisher), ethyl acetate (Fisher), hexane (Fisher), 7 M ammonia in methanol (Thermo Fisher), 1 M hydrochloric acid (Fisher), diethyl ether (Fisher), 50% w/v sodium hydroxide (Fisher).

### Synthesis and Spectroscopic Characterisation of SalACP

SalACP was synthesised from salicylaldehyde in one step using 4,4,5,5-tetramethyl-2-(3-methylbut-2-en-1-yl)-1,3,2-dioxaborolane and ammonia (Scheme S1). For the synthetic procedure, see the Supplementary Information. Characterisation data is as follows (for numbering, see Scheme S1):

^1^H NMR (400 MHz, DMSO-d_6_) δ 7.04 (1H, ddd, *J* = 8.1, 7.4, 1.7 Hz, 2-CH), 6.98 (1H, dd, *J* = 7.6, 1.7 Hz, 6-CH), 6.68 (1H, app td, *J* = 7.4, 1.3 Hz, 1-CH), 6.64 (1H, dd, *J* = 8.1, 1.2 Hz, 3-CH), 5.90 (1H, dd, *J* = 17.5, 10.8 Hz, 9-CH), 5.03 (1H, d, *J* = 1.4 Hz, 10-CH_2_cis), 5.02 (1H, dd, *J* = 28.5, 1.5 Hz, 10-CH_2_trans), 3.91 (1H, s, 7-CH), 0.94 (3H, s, 11-CH_3_), 0.93 (3H, s, 12-CH_3_). ^13^C NMR (100 MHz, DMSO-d_6_) δ 158.7 (4-C), 146.5 (9-C), 130.9 (5-C), 128.2 (2-C), 125.3 (6-C), 117.7 (1-C), 116.7 (3-C), 113.35 (10-C), 62.2 (7-C), 42.6 (8-C), 24.9 (11-C), 21.1 (12-C). MS (ES +) m/z 176 [M-NH_2_]^+^. HRMS (ES +) [M-NH_2_]^+^ C_12_H_15_O requires 175.1124, found 175.1123.

### NMR Experiments

NMR experiments were conducted on a Bruker 500 MHz spectrometer equipped with a 5 mm BBO probe and TopSpin 4.0.1 software. Samples were generally prepared in microcentrifuge tubes before being transferred to Norell NMR tubes for analysis. Experiments were conducted at 25 °C with water suppression. Data processing was conducted in Topspin 4.1.4 software.

### SPME–GC–MS Experiments

#### General

SPME–GC–MS experiments were conducted on an Agilent 8890 GC coupled to a Leco Pegasus BT4D time-of-flight mass spectrometer (LECO Instruments UK) fitted with a CTC-PAL3 auto-sampler (JSB). Samples were pre-incubated for 10 min at 500 rpm at either 80 °C (head-space) or 30 °C (immersive) before extraction with the SPME Arrow fibre (Carbon WR/PDMS, 1.1 mm × 120 μm, Agilent Technologies), which was pre-conditioned (300 °C for 5 min) before addition to the sample vial (8 mL, Agilent Technologies). Extraction was conducted with 20 mm vial penetration (head-space) or 40 mm vial penetration (immersive) for 40 min. Desorption into the GC inlet was conducted at 250 °C for 3 min before column separation (DBWAX capillary column, 30 m × 250 μm × 0.25 μm, Agilent Technologies). For immersive extraction, the fibre was agitated in water for 5 min between samples.

#### GC conditions

For experiments with cysteamine, an initial column temperature of 80 °C was used, which was raised to 150 °C at a rate of 7 °C min^−1^, then to 220 °C at a rate of 30 °C min^−1^. The total GC run time was 17 min and 20 s per sample. For experiments with SalACP, an initial column temperature of 100 °C was used, which was raised to 150 °C at a rate of 7 °C min^-1^, then to 240 °C at a rate of 40 °C min^−1^. The temperature was then held at 240 °C for 4 min. The total GC run time was 13 min and 24 s per sample. The GC inlet was operated in split mode at ratios of 7:1 (cysteamine) or 20:1 (SalACP). Helium was used as the carrier gas at a flow rate of 1 mL min^-1^. The septum purge rate was 3 mL min^−1^.

#### MS

A scan range of 45–200 amu was used with an extraction frequency of 32 kHz, and acquisition rate of 10 spectra per second. For cysteamine, the ion at *m/z* 89 was used for quantification (retention time = 420–422 s) and was compared to the ion at *m/z* 90 corresponding to the internal standard (^13^C-labelled thiazolidine, retention time = 420–422 s). For samples with salicylaldehyde, the ion at *m/z* 122 was used for quantification (retention time = 440–442 s) and was compared to the ion at *m/z* 105 corresponding to the internal standard (acetophenone, retention time = 420–422 s).

#### Data analysis

Data was acquired using ChromaTOF (LECO Instruments UK). Numerical analysis was conducted in Microsoft Excel and GraphPad Prism software.

#### Human cell culture

HepG2 cells were grown in T75 or T125 tissue culture flasks with vented lids. Media consisted of Minimum Essential Medium Eagle (MEM) media containing Earle’s salts, L-glutamine, sodium bicarbonate, 10% foetal bovine serum, sodium pyruvate and Pen-Strep. Cells were incubated at 37 °C in a humidified atmosphere (5% CO_2_) and were grown to 80% confluency before passaging, which was conducted after treatment with 0.05% trypsin with ethylenediaminetetraacetic acid (EDTA) in phosphate-buffered saline. After 5 min treatment at 37 °C, 1 mL of media was added before the cell were pelleting by centrifugation and then resuspended in media and seeded in a new flask.

## Supplementary Information

Supplementary Information.

## Data Availability

All data relevant to the work presented are available in the main article and Supplementary Information. All data are available for the corresponding authors on reasonable request.
