# Supplementary material for: Formaldehyde quantification using gas chromatography–mass spectrometry reveals high background environmental formaldehyde levels
Source: Sci Rep. 2024 Sep 4;14:20621. doi: 10.1038/s41598-024-71271-z (PMC11375156; doi:10.1038/s41598-024-71271-z)
Supplement: Supplementary file 1 — Supplementary Information. [file 41598_2024_71271_MOESM1_ESM.pdf]

Supplementary Information

Formaldehyde quantification using gas chromatography-mass spectrometry reveals high background environmental formaldehyde levels

Sara Y. Chothia[1], Vicki L. Emms[1], Liam A. Thomas[1], Natasha F. A. Bulman[1], Paul S. Monks[2], Rebecca L. Cordell[2*], Richard J. Hopkinson[1*]

*[1]Leicester Institute for Structural and Chemical Biology and School of Chemistry, University of Leicester, Henry Wellcome Building, Lancaster Road, Leicester, LE1 7RH, UK. *E-mail: rc145@leicester.ac.uk; richard.hopkinson@leicester.ac.uk*

[2]Space Park Leicester, University of Leicester, 92 Corporation Road, Leicester, LE4 5SP, UK

Supplementary Figures

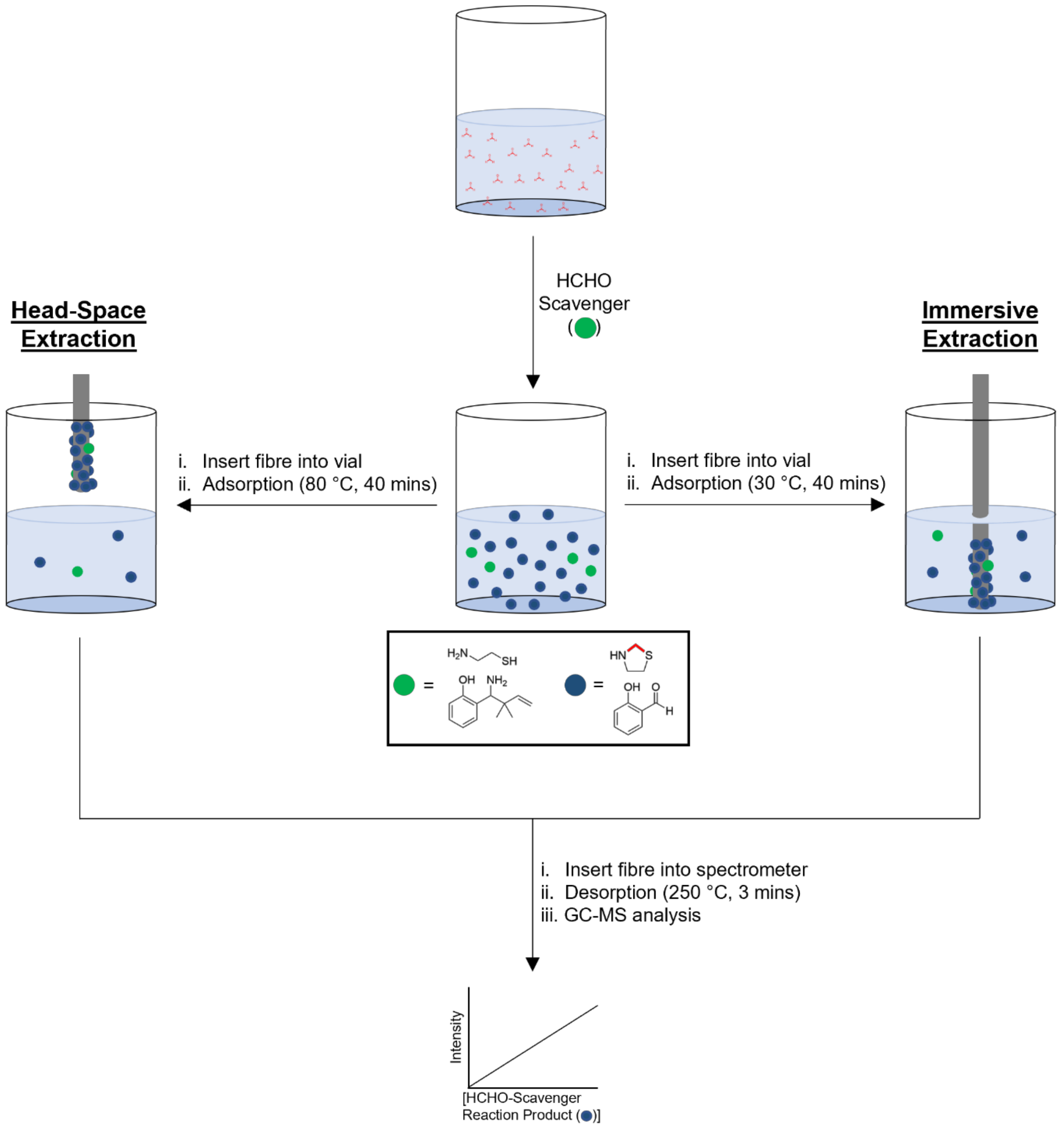

Figure S1. Scheme showing head-space and immersive SPME-GC-MS method workflows. Formaldehyde (HCHO) scavengers (either cysteamine or SalACP, green circles) are added to the aqueous samples and left to react with HCHO (red). The SPME fibre is then inserted into the sample vials. For head-space extraction, the fibre is positioned above the liquid sample, while the fibre is inserted into the liquid during immersive extraction. Heating the samples induces adsorption of the scavenger-HCHO reaction products (thiazolidine or salicylaldehyde, blue circles) onto the fibres, which are then removed from the vials and transferred to the mass spectrometer. The reaction products are then desorbed from the fibres and analysed by GC-MS.

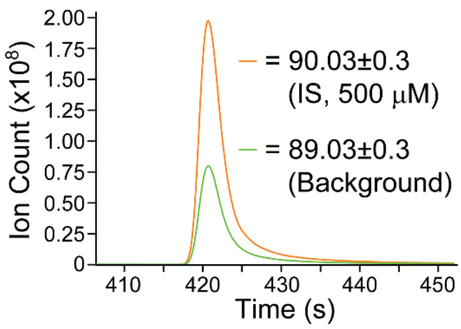

Figure S2. Image from a GC chromatogram from a sample of HEPG2 cell lysate in 100 mM sodium phosphate buffer pH 7.4 without added HCHO after head-space extraction (green). A peak corresponding to HCHO-derived thiazolidine is observed (green). A peak corresponding to the internal standard (IS, ^{13}C-labelled thiazolidine) is shown for reference (orange).

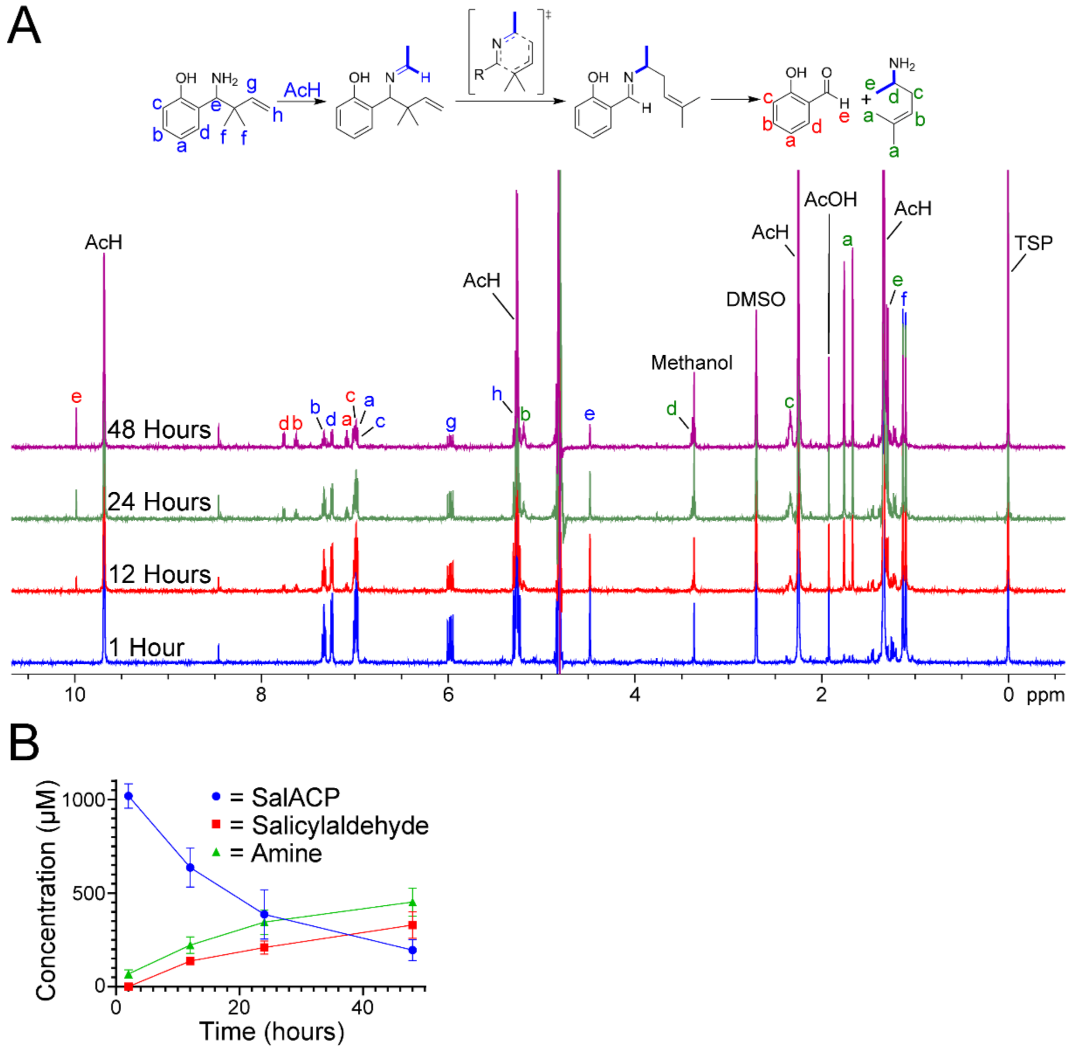

Figure S3. (A) ^{1}H NMR spectra of a sample of SalACP (1.67 mM) incubated with acetaldehyde (16.7 mM) at 25 °C. Resonances corresponding to SalACP, salicylaldehyde and amine product are highlighted. (B) Graph showing time-dependent formation of salicylaldehyde and amine from the reaction of SalACP and AcH (concentrations calculated from experiments in (A)).

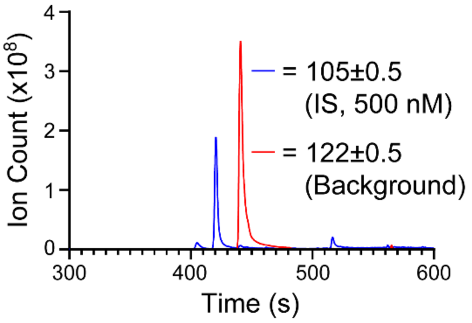

Figure S4. Image from a GC chromatogram from a sample of SalACP in 100 mM sodium phosphate buffer pH 7.4 without added HCHO after immersive extraction (blue). A peak corresponding to salicylaldehyde is observed (red). A peak corresponding to the internal standard (IS, acetophenone) is shown for reference (blue).

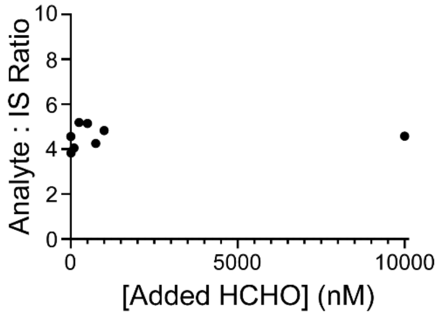

Figure S5. Graph showing quantification of salicylaldehyde derived from the reaction of SalACP and HCHO (1 nM – 10 μM) in 100 mM sodium phosphate buffer pH 7.4 using head-space SPME GC-MS. No dose-dependent response is observed.

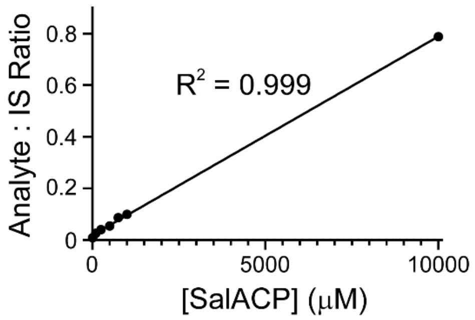

Figure S6. Graph showing quantification of salicylaldehyde derived from the reaction of SalACP (10 μM – 10 mM) and HCHO (500 nM) in 100 mM sodium phosphate buffer pH 7.4 using head-space SPME GC-MS. A linear response is observed (R^2 = 0.999).

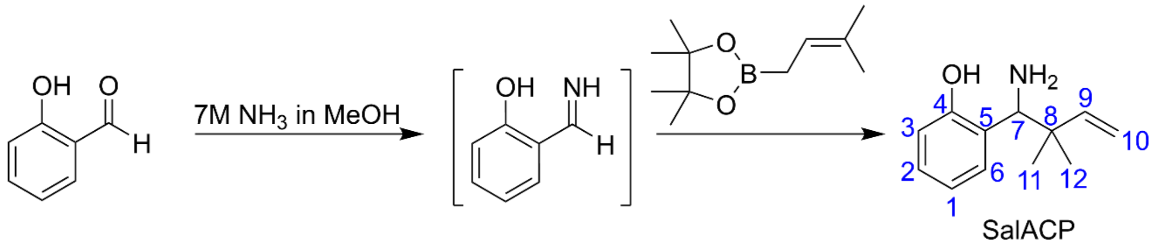

Scheme S1. Synthetic route to SalACP.

Synthesis of SalACP

Salicylaldehyde (400 μL, 0.468 g, 3.83 mmol) was added to 7M ammonia in methanol (10 mL) and cooled in an ice bath. After stirring at 0 °C for 1 hour, 4,4,5,5-tetramethyl-2-(3-methylbut-2-en-1-yl)-1,3,2-dioxaborolane (0.847 g, 1.13 equiv.) was added dropwise to the ice-cold mixture. The mixture was allowed to warm to room temperature and then heated overnight under reflux. Ammonia was purged from the reaction mixture under a stream of nitrogen gas and the resulting mixture was evaporated to dryness under reduced vacuum (without heat). The crude product was redissolved in dichloromethane (10 mL) and washed with water (3 x 10 mL). The organic extract was dried over anhydrous sodium sulfate and dried *in vacuo*. Residue was dry-loaded onto silica gel and purified by flash column chromatography (30 % ethyl acetate in hexane) to give a pale yellow solid (0.607 g, 3.18 mmol, 83 %).
